# Utilising primary care electronic health records to deliver the ALABAMA randomised controlled trial of penicillin allergy assessment

**DOI:** 10.1186/s13063-024-08506-x

**Published:** 2024-10-03

**Authors:** Shadia Ahmed, Joanne Fielding, Catherine E. Porter, Kelsey F. Armitage, Marta Wanat, Chris Bates, Lazarina Engonidou, Robert M. West, Ly-Mee Yu, Ushma Galal, Philip Howard, Christopher C. Butler, Sinisa Savic, Jenny Boards, Sarah Tonkin-Crine, John Parry, Sue A. Pavitt, Jonathan T. Sandoe

**Affiliations:** 1 Leeds Institute of Medical Research, University of Leeds, Leeds, UK; 2 Department of Microbiology, Leeds Teaching Hospitals NHS Trust, Leeds, UK; 3 Dental Translational and Clinical Research Unit, School of Dentistry, University of Leeds, Leeds, UK; 4 The Nuffield Department of Primary Care Health Sciences, University of Oxford, Oxford, UK; 5 The Phoenix Partnership, Leeds, UK; 6 Leeds Institute of Health Sciences, University of Leeds, Leeds, UK; 7 North-East Yorkshire, NHS England, Newcastle, UK; 8 School of Healthcare, University of Leeds, Leeds, UK; 9 Leeds Institute of Rheumatic and Musculoskeletal Medicine, University of Leeds, Leeds, UK; 10 SMILE AIDER PPI Group, Leeds Clinical Research Facility, The Leeds Teaching Hospitals NHS Trust, Leeds, UK

**Keywords:** Penicillin allergy, Electronic health record, EHR, Primary care, SystmOne, TPP, RCT, Clinical trials, EMIS, Penicillin allergy de-labelling

## Abstract

**Background:**

Use of electronic health records (EHR) to provide real-world data for research is established, but using EHR to deliver randomised controlled trials (RCTs) more efficiently is less developed. The Allergy AntiBiotics And Microbial resistAnce (ALABAMA) RCT evaluated a penicillin allergy assessment pathway versus usual clinical care in a UK primary care setting. The aim of this paper is to describe how EHRs were used to facilitate efficient delivery of a large-scale randomised trial of a complex intervention embracing efficient participant identification, supporting minimising GP workload, providing accurate post-intervention EHR updates of allergy status, and facilitating participant follow up and outcome data collection. The generalisability of the EHR approach and health economic implications of EHR in clinical trials will be reported in the main ALABAMA trial cost-effectiveness analysis.

**Methods:**

A descriptive account of the adaptation of functionality within SystmOne used to deliver/facilitate multiple trial processes from participant identification to outcome data collection.

**Results:**

An ALABAMA organisation group within SystmOne was established which allowed sharing of trial functions/materials developed centrally by the research team. The ‘ALABAMA unit’ within SystmOne was also created and provided a secure efficient environment to access participants’ EHR data. Processes of referring consented participants, allocating them to a trial arm, and assigning specific functions to the intervention arm were developed by adapting tools such as templates, reports, and protocols which were already available in SystmOne as well as pathways to facilitate allergy de-labelling processes and data retrieval for trial outcome analysis.

**Conclusions:**

ALABAMA is one of the first RCTs to utilise SystmOne EHR functionality and data across the RCT delivery, demonstrating feasibility and applicability to other primary care RCTs.

**Trial registration:**

ClinicalTrials.gov: NCT04108637, registered 05/03/2019. ISRCTN: ISRCTN20579216.

**Supplementary Information:**

The online version contains supplementary material available at 10.1186/s13063-024-08506-x.

## Background

Electronic health records (EHR) provide real-world data that are increasingly being interrogated to advance retrospective, observational clinical research. Large-scale EHR data mining has become increasingly popular for prognostication, comparative effectiveness explorations, and population health [1]. However, these study designs can identify associations but cannot entirely control for confounding. Embedding prospective randomised controlled trials (RCTs) in EHR systems has progressed more slowly but holds great potential for improving recruitment, applicability of findings, trial efficiency, and reducing bias [2]. The vast majority of RCTs using EHR have evaluated EHR-based interventions, such as alerts or other clinical decision–support systems [3]. Another frequently utilised function is to aid recruitment by facilitating preliminary eligibility screening and efficient participant identification [3]. Attempts have been made to ascertain clinical outcome data from EHR, replacing the often onerous data collection processes in traditional RCT, but this has not always been successful and there are a number of challenges to utilising EHR in research [1, 4].

RCTs utilising primary care EHR data are few [5]. Here, we describe how we have delivered the Allergy AntiBiotics And Microbial resistAnce (ALABAMA) (ClinicalTrials.gov: NCT04108637, ISRCTN20579216) RCT using EHRs to identify and screen participants, to facilitate participant follow-up, to collect outcome data, and to update records based on trial interventions. ALABAMA was a pragmatic, 1:1, open label, RCT evaluating a penicillin allergy assessment pathway vs usual clinical care in a UK primary care setting [6, 7]. Penicillin allergy is common, affecting approximately 6% of the population, and is associated with many harms including worse health outcomes, infection with resistant pathogens, and increased exposure to antibiotics [9, 10]. The rationale for the trial relates to the high prevalence of patients labelled as penicillin allergic and the fact that most of these patients do not have true hypersensitivity when they are formally tested [8]. The trial aimed to determine if allergy records can be safely removed, if penicillin prescribing can resume, and the impact on patient health outcomes. The ALABAMA trial was conducted in 51 NHS general practices across England, and trial oversight was carried out by the University of Leeds and the Primary Care Clinical Trials Unit at the University of Oxford.

SystmOne is a centralised cloud based EHR developed by The Phoenix Partnership (TPP). A key feature of SystmOne is its ability to provide the controlled sharing of patient records for direct care. It is used by over 9000 organisations in the UK and internationally, including approximately 40% of general medical practices in England [11]. We aimed to utilise SystmOne to facilitate a number of ALABAMA trial processes. Analytical functionality in SystmOne facilitates the creation and execution of queries via generated reports at a population level. Therefore, it was possible to identify potential trial participants who had a penicillin allergy record using this functionality. We wanted to be able to use real time antibiotic prescribing data to prompt participant follow-up, rather than rely on general practitioners to remember to collect follow-up data. In line with UK efforts to deliver more efficient RCTs [12], ALABAMA incorporated data linkage and collection of secondary outcome measures from NHS England healthcare databases (Hospital Episode Statistics and Office for National Statistics) [6, 7]. The ALABAMA research team developed innovative methods of recruitment and delivery of the intervention as well as real time follow-up and data collection largely using existing functions available within SystmOne.

Whilst healthcare professionals remain supportive of clinical research, their high workload can be a significant and unavoidable barrier to trial participation. The additional burden that can be introduced by trial requirements—over and above routine clinical practice—can prohibit participation. This is certainly true of general practitioners in the NHS, due to their high workload pressures. We therefore wanted to design a trial that would require minimal input from general practitioners in terms of research delivery as well as making trial processes more efficient and data collection more reliable and to pave the way for optimising the use of routinely collected clinical data in primary care RCTs. The aim of this paper is to describe the development and implementation of these novel processes for trial delivery as well as how safe management of participant data has been achieved. We believe the principles of these innovative methods are promising and path the way for utilising an EHR approach to efficient and safe clinical trial delivery and outcome collection.

## Method

The ALABAMA trial was approved by the London Bridge Research Ethics Committee (ref: 19/LO/0176) and recruitment took place between September 2019 and October 2023. The ALABAMA trial protocol has been published in full previously [6, 7]. The trial flow diagram is available in the published protocol [6, 7], and participant eligibility criteria can be found in the supplementary material. General practitioner (GP) sites were approached for participation if they used SystmOne and were located near an ALABAMA secondary care site where the ALABAMA intervention was delivered; 113 sites expressed an interest to participate of which 51 sites recruited [6, 7]. The ALABAMA primary outcome was the proportion of participants receiving penicillin prescriptions for predefined conditions where a penicillin is the first-line recommended antibiotic up to 12 months post randomisation. Development of the EHR SystmOne Clinical Development Kit (CDK) functions used for the ALABAMA trial was a collaborative, co-design process between the ALABAMA research team and TPP clinical, technical, and research teams. Patient and public involvement and engagement (PPIE) was incorporated using the SMILE AIDER PPIE Forum with early exploration of acceptability of using EHR for the ALABAMA RCT and gaining insight in how to explain this approach to potential participants. SMILE AIDER was not involved in the technicalities of building functionality in TPP; this was facilitated by TPP product specialists who are SystmOne experts and who helped the ALABAMA research team to adapt configurable functionality within it for use in a trial setting. Two members of the ALABAMA research team (JF and JS) worked with TPP trainers to develop SystmOne functionality prior to the start of the trial. JF and JS are competent users of basic computer applications but had no prior experience using SystmOne. JS and JF had approximately 5 h of face-to-face training/assistance with development work; thereafter, the helpdesk provided ad hoc support. Ongoing discussions with patient and public contributors as well as a general practitioner advisory group took place throughout the process, through attendance at meetings or SMILE AIDER meetings. The trial steering committee (TSC) was informed during the process and kept updated via formal TSC meetings. A SystmOne development environment, which has full functionality but only contains data for fictitious patients, was used to build and test the ALABAMA trial functions before being replicated and tested in the live, production version of SystmOne. Work was broadly organised into two sections: (1) trial processes and (2) data protection and information governance. Penicillin allergy de-labelling tools and procedures will be described in full in a separate publication.

## Trial processes

### Process mapping

**Fig. 1:**
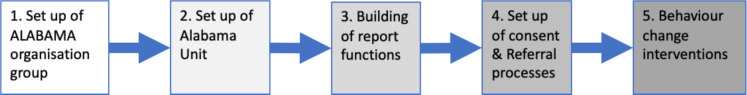
ALABAMA trial components agreed as suitable for construction in SystmOne

Initially, a flow diagram was drafted by the ALABAMA clinical lead to encompass each element of the trial pathway from participant identification through to follow-up; this included a list of standard operating procedures (SOP) or working instructions (WI) needed for each trial process and defined who would be undertaking each task during trial delivery (Fig. S1). The technical co-design approach between the ALABAMA research team and TPP staff was iterative; after generating an initial draft flow diagram, a final version was reached by consensus. The final version was annotated with details of the research team, task, and location for each trial action. Details of what actions the research team member would have to perform and whether functionality in SystmOne could potentially be utilised were agreed and documented. Feedback from the wider team and progress updates were provided during weekly team meetings and monthly trial management group (TMG) meetings. The research team and TPP decided together which of the potential trial processes were suitable to be delivered using SystmOne and what existing functionality would be best to deliver each process. This information was added to the flow diagram to create a work list. The ALABAMA research team, who did not have prior experience using SystmOne, then worked with TPP product specialists to build each component based on the chronology of the participant journey (Fig. 1). This extensive preparative stage took several months to execute.

## Set up of the *ALABAMA* ‘organisation group’ in SystmOne

An** ‘**organisation group’ is a virtual collaboration group within SystmOne. It allows participating organisations (e.g. general practices) to share and subscribe to Clinical Development Kit content. Such content includes documents, reports, templates, and protocols (see later). An ‘ALABAMA organisation group’ was set up centrally by TPP.

## Set up of the ‘*ALABAMA* unit’ in SystmOne

An ‘ALABAMA unit’ was created using existing SystmOne functionality, in the same way that a new general practice might be set up on SystmOne. Creation of the ALABAMA unit enabled the research team to utilise the existing data entry and protection rules built into SystmOne, as the unit had equivalent data safety features as a new ‘virtual’ general practice. The set up was administered centrally at TPP.

## Utilisation of the *ALABAMA* unit

The controlled secure sharing of patient level EHR data within SystmOne between the general practice held record and the ALABAMA unit enabled the researchers to access patient level data real time without complex data extraction procedures. Patient progress through the trial could be monitored including the removal of penicillin allergy labels by general practitioners where appropriate. The ALABAMA unit provided a secure location into which consented participants could be electronically ‘referred’ and their general practice EHR accessed by the research team. Access was restricted to appropriate members of the ALABAMA research team and informed consent from participants had to be obtained prior to referral into the ALABAMA unit.

Templates, reports, and protocols are Clinical Development Kit (CDK) tools that exist within the SystmOne EHR; they facilitate record keeping and delivery of care. These tools were utilised to allow documentation and delivery of trial related activities. Templates allow the standardisation and optimisation of data entry into EHRs. Reports are configurable functions that allow detailed queries to be executed on a complete GP practice population, for example reports can be used to identify patients who fulfil trial inclusion criteria. Protocols are configurable functions that support clinical and administrative pathways and user-defined decision support.

Publishing functions in the ALABAMA SystmOne unit enabled templates, reports, and protocols to be shared with participating general practices via the ALABAMA organisation group. This centralised, real-time content sharing functionality is a core aspect of the TPP CDK.

Participant and data flow at each stage of the trial was identified and mapped then subdivided into “function units” which were approached as distinct components (Fig. S1). These were drafted by JS and modified iteratively during discussions within the ALABAMA research team and with TPP and the trial team and are broadly summarised into the following key components:

1. Site set up
2. Participant screening and invitation processes
3. Eligibility check, consent, and referral
4. Randomisation, baseline data collection
5. Penicillin allergy testing and reporting of results
6. Antibiotic prescription identification
7. Trial outcome reports

## Templates

Templates in SystmOne facilitate data entry within a patient record, allowing for data to be entered in a controlled manner using tick boxes and data entry fields. Templates were created for ALABAMA for the purposes of participant referral into the ALABAMA unit, to allow assignment of participants into a trial arm and to enter test results into participants’ records.

## Reports

Clinical reporting in SystmOne is a logical step-wise process where report criteria are built in sub-reports then linked together. Reporting functionality was used to identify potential trial participants, to aid trial progression and to check for participants who had received a recent antibiotic prescription. Once reports were built and tested, they were published to the ALABAMA unit so that they were visible to any GP sites that had been invited to be members of the ALABAMA organisation group. Each new report was named and assigned a category and sub-category in SystmOne. New report categories were created for ALABAMA so they would be easily identifiable; these included ALABAMA GP reports to run, ALABAMA research nurse reports, and ALABAMA trial outcome reports. These report categories were used for participant screening, follow-up, and outcome data collection respectively. All reports were built from scratch, which included creating over 40 sub-reports that were then joined as required. For example, to create the patient screening report used to identify potential eligible participants, key inclusion and exclusion criteria were included to create sub-reports which were then joined to create the final screening report. This report was amended following any approved protocol changes.

To monitor and follow up participants, TPP designated an existing numerical field to record the participant trial ID number. Reports were created which identified trial ID numbers and then combined with other sub-reports in order to extract the required data. For example, to identify participants awaiting randomisation, a sub-report was created that identified participants not yet randomised.

## SystmOne protocols

Protocols within SystmOne are used to help with workflow and support decision making from within a patient record. These can be basic or complex and can be used as reminders for specific tasks. Protocols can also be used in the background of a template to carry out an action. For the ALABAMA trial, protocols were devised in development sessions to assist with entering results and clinical codes for allergy testing. Protocols were used to create GP pop-up messages which appeared during consultations, asking GPs to remind trial participants to complete a symptom diary if prescribed an antibiotic during the trial follow-up period. Protocols were also developed to aid confirmation of trial ID numbers after referral to the ALABAMA unit, trial arm assignment, and removal of allergy labels. The caseload function in SystmOne allows separation of work streams and was utilised to allocate participants into trial arms following randomisation. Caseloads were used for this purpose as a participant can only be assigned one caseload per referral and therefore allowed documentation of trial arm assignment within the participant’s electronic health record.

## Testing functionality and training

SOPs/WI were written for all trial processes involving use of SystmOne. These were provided to general practices and the research team during training sessions. The SOPs/WI were subsequently updated in response to feedback and modifications to trial processes.

## Data protection and information governance

The ALABAMA trial was designed to be compliant with data protection regulations as outlined in the study protocol [6, 7]. Data protection and information governance measures were co-developed and refined during the set-up phase of the trial and involved consultation with a range of stakeholders (e.g. GP advisors, the former Leeds National Health Service (NHS) Clinical Commissioning Group, Leeds Institute of Data Analytics and the Data Protection Officer at the University of Leeds). The research team was required to produce a Data Protection Impact Assessment (DPIA) [13] which was approved by the University of Leeds Secretariat. The DPIA process helped the research team to identify and minimise data protection risks associated with access to, and processing of, special category personal data to comply with the General Data Protection Regulation (GDPR).

## Results: how SystmOne tools were utilised in the *ALABAMA* trial

### Trial processes

#### Site set up

TPP set up an ‘ALABAMA organisation group’ which successfully allowed GPs involved in the research to access trial materials/functions. All 51 general practices taking part in the trial were ‘invited’ to be part of this organisation group (staggered over time), accepted this invitation locally, and were able to use ALABAMA trial materials/functions. The ALABAMA unit was set up by TPP, and data sharing agreements and processes were put in place between TPP and the University of Leeds (RCT sponsor). The ALABAMA unit served as a shared system between the research team and enrolled general practices for the purposes of the trial. Each stage of the trial utilised SystmOne functions, which were accessed via the ALABAMA unit. An overview of participant flow and required functions is shown in Fig. S1; SOPs/WI were completed for all aspects of the trial delivery (see Fig. S1).

#### Participant screening and invitation processes

A screening report was built to identify potentially eligible patients; this included patients over the age of 18, with a penicillin allergy listed in their records and who had been prescribed antibiotics in the 12 months prior to screening. The report was ‘published’ to the organisation group, and all 51 participating general practices could run the report locally restricted to their own practice patient population and invite patients to participate, as data controller (patients listed in the SystmOne screening report were contacted via an invitation letter, together with an ‘expression of interest’ form to return if appropriate). A total of 1506 of the 94,599 (1.6%) patients registered at the 11 general practices enrolled in the feasibility study were identified as potentially eligible. A small number of false positive screening results (i.e. ineligible patients) were identified during the feasibility study which was resolved by refining the report (see supplemental material). Whilst the report could capture all inclusion criteria, certain exclusion criteria required manual checking as the data recorded in the EHR records was not considered reliable enough, for example information on a whether a patient was pregnant was not always up to date in the EHR (see supplemental material for details). Inclusion criteria were modified after the feasibility study to allow a greater window for prior antibiotic treatment to 24 months. In this period, 16,419 of the 597,071 (2.75%) patients registered at participating general practices were identified as potentially eligible, and 70% of those were invited to take part. The discrepancy was due to GPs pre-screening for potential exclusion criteria before mail out to identify those who did not meet the eligibility criteria. However, this step was removed in a protocol amendment as it was felt to be unnecessary burden on general practitioners, as potential participants underwent a further thorough review and identification of any exclusion criteria at an eligibility and consent appointment.

#### Eligibility check, consent, and referral

When patients expressed an interest in participation, consent was arranged with the GP (or a trained delegate), either face to face or via telephone. Paper/online consent forms were annotated/printed with unique trial identification (trial ID) numbers. Once consent had been obtained (which included consent to share their EHR), participants were then ‘referred’ to the ALABAMA unit using a ‘consent and referral’ electronic template built in SystmOne (Fig. S2). This template allowed the GP to record that the participant had consented to the trial and that the GP (as the data controller) also agreed that the EHR could be shared to the ALABAMA unit. There is no functionality currently in SystmOne to auto-allocate a unique trial identification number to trial participants, so the participant’s trial ID number was entered manually into the template, and the participant was referred into the ALABAMA unit with a single click. All new referrals into the ALABAMA unit were subsequently picked up and accepted by the research team who looked Monday–Friday for referrals using the “Tasks” function within SystmOne. Participants were monitored using a ‘Consented Patients’ report which was created to identify participants assigned a trial ID number greater than 0 and coded to have been consented into the trial. An “ALABAMA View” template was created in SystmOne to assist the research team checking the participant’s EHR. This was a “window” into the health record that displayed live data pertinent to ALABAMA eligibility criteria such as recent medication and allergy records.

#### Randomisation and baseline data collection

Reports were written to generate an online view of all consented participants. A ‘Referrals Awaiting Baseline visit’ report was built to monitor referral of new participants into the ALABAMA unit and identify those awaiting a baseline assessment and randomisation. The report was built by combining two reports, one which identified participants who had a clinical trial ID number and the other identified ‘caseload’ (trial arm). These reports were then combined to identify participants with a trial ID number who did not have a caseload (trial arm) assigned. Once participants were randomised to a trial arm via a separate online randomisation system (Sortition) provided by the Primary Care Clinical Trials Unit at the University of Oxford, the research team could then manually record whether the participant was assigned to usual care or the intervention arm in SystmOne via a template which used the ‘caseload holder function’. This enabled selective delivery of trial and behaviour change materials [14] depending on the trial arm allocation (Fig. S3). Behaviour change materials included a clinician leaflet (‘Penicillin Allergy Testing: Information for general practice’) and two patient booklets (‘Penicillin Allergy Testing: going for a test’ and ‘Penicillin Allergy Testing: a negative test result’). Baseline data collection took place via telephone interview with the participant, and data were entered manually into a traditional trial database (OpenClinica).

#### Penicillin allergy testing and reporting of results

Participants randomised to the intervention arm underwent penicillin allergy testing at a secondary care hospital clinic. An ALABAMA penicillin allergy test result template was designed to facilitate the correct results being entered into a participant’s record by GP practice staff following receipt of the test results (Fig. S4). There were no CTV3 or SNOMED Clinical teams codes [15] to describe a patient having had penicillin allergy testing and the results, so bespoke “y” codes were written into SystmOne to allow accurate coding of the test and results (Table S1). To aid removal of allergy labels when participants tested negative following penicillin allergy testing, ‘pop up’ reminders (if not deactivated) and tasks were used to remind GPs (or their authorised delegate) to update the record allergy status, which were linked to the testing “Y” codes and results, to ensure only those with appropriate results received ‘pop-ups’.

#### Antibiotic prescription identification

During follow-up, the research team used the SystmOne report function to generate a daily report of antibiotic prescriptions so that trial outcome data including data related to the primary outcome could be collected. This report triggered the research team to contact the participant to collect more detailed information about the infection episode and prescription for predefined infections [6, 7]. The report was built by identifying participants with a trial ID number, who had been allocated to a caseload (trial arm) and prescribed antibiotics in the previous 5 days.

#### Trial outcomes

In order to capture data regarding the primary outcome using coded (SNOMED CT) indications for antibiotic prescriptions as well as prescription data, and penicillin allergy de-labelling data, TPP supplied a centralised bespoke research-level extract for all consented participants tailored to the ALABAMA trial. Some additional data processing was needed before analysis for some variables, for example, antibiotic dosage and frequency are currently recorded as textual strings by GPs in the English NHS, and so these needed to be processed in order to obtain numeric values for research analysis [16].

## Data protection and information governance

The research team was able to access the EHR of consented participants via the ALABAMA unit and control who was able to view those records. In line with normal data protection practice in SystmOne, each participating GP practice was the data controller for its own data. As sponsor of the ALABAMA trial, the University of Leeds acted as the data controller of the ALABAMA unit created in SystmOne. In order for trial participants to be electronically referred into the ALABAMA unit in SystmOne, TPP required the University of Leeds to sign a processing agreement before they could turn on the SystmOne data sharing functionality. The processing agreement required signature by the University’s Data Protection Officer.

The ALABAMA research team set up data processing agreements (part of a model Non-Commercial Agreement for Research) between the University of Leeds and participating hospital sites (where the penicillin allergy testing was delivered). Similarly, a data sharing agreement was implemented between the Universities of Leeds and Oxford (trial co-ordination centres) and the Leeds Teaching Hospitals NHS Trust (NIHR grant holder and principal testing site). In order to comply with TPP’s data security requirements, the research team could only access SystmOne via a secure N3/Health and Social Care NHS network (HSCN). Research team members with access to SystmOne were required to have NHS employment contracts (either substantive, or honorary for University of Leeds staff) and appropriate data protection and information governance training.

A pseudonymised, encrypted SystmOne bespoke data extract was transferred to the University of Oxford’s Primary Care Clinical Trials Unit (PC-CTU) via a secure file transfer protocol (FTP). Only data required for appropriate trial analysis was collected and this is detailed in a Data Management Plan managed by the University of Oxford PC-CTU. These measures, together with the informed consent of trial participants, protect their rights and ensured that the trial was compliant with the requirements of the GDPR.

## Discussion

ALABAMA demonstrates the feasibility of incorporating an EHR system into multiple aspects of the delivery of an RCT; including participant identification, intervention delivery, follow-up, and outcome data collection. This was done by adapting clinical tools (protocols, templates and reports) that already existed within the SystmOne EHR to allow delivery of the trial and facilitate collection of outcome data. This collaborative research co-designed with the EHR provider is novel and gives confidence in achieving the goal of efficient RCT delivery using routinely collected data. This is a descriptive report of novel trial methodology, which was achieved using predominantly existing functionality within the SystmOne EHR system, i.e. without expensive de novo IT development work. The project was undertaken to minimise the time required by GPs to participate in trial delivery, to optimise trial processes and quality of data collection, and to pave the way for trial design and delivery to be built into routine point of care EHR systems. It demonstrates that it was possible to streamline many trial processes making trial delivery more efficient. The reduced need for direct participant contact afforded by the novel EHR-supported design was seen as reducing the burden to participants and GPs taking part and attractive to the NHS Research Ethics Committee approving the trial. Real-time identification of antibiotic prescriptions was achieved in the trial using a built in EHR prescribing report which prompted research nurse follow-up; this was probably more effective than the traditional alternative method of asking GPs to complete case reports forms (CRFs), although we did not carry out a direct comparative evaluation. There are likely to have been over 500 prescribers across our 51 participating general practices, and each would have needed to be trained, remember to collect primary event data, and complete CRFs, when they prescribed antibiotics to a trial participant. Data capture through the EHR is therefore likely to have been more accurate and complete.

Although there is a current desire to enhance the use of routinely collected clinical data in RCTs in the UK [12], a recent systematic review found relatively few RCTs used EHR, with most studies coming from the USA and just 5% from the UK [3]. In terms of five previously reported challenges to using routine EHR data in RCTs, we encountered the following [1]:

1. In terms of data not being organised for research purposes [1], this was evident in the extracted antibiotic prescribing dosage data that required further processing;
2. Whilst data are both densely and irregularly observed, we overcame this problem by having objective outcome measures and an analysis plan that determined how such events should be handled;
3. Not all data elements that we wanted were recorded in the EHR, for example we needed information on the duration of infection symptoms which had to be collected from participants via a symptom diary;
4. The fact that EHR data are both cross-sectional and longitudinal was not a major problem but was evident, for example, in the reliability of the date of penicillin allergy diagnosis, which may have been migrated from other EHR systems or from paper records;
5. The fact that information collected in EHR is informative, i.e. data are biased towards sicker people, was not a problem as we had objective outcomes and a robust method of following up via the ALABAMA unit.

Others have found additional challenges, particularly consolidating data across different EHR systems [4], which we avoided by only recruiting from general practices using SystmOne, arguing that if we can demonstrate feasibility with one EHR provider, we can then expand and engage others in future work.

The development of the ALABAMA unit and its related templates, protocols, and reports was a lengthy process that required close collaboration with the TPP team as it had not been done before in the context of an RCT. Prior to ALABAMA, the research team had limited experience with SystmOne, and therefore the input from TPP staff and a convened expert GPs group was crucial. Working with GPs, who were familiar with SystmOne and the ALABAMA unit, facilitated feedback that allowed the research team to make iterative changes and improve processes. The template, protocol, and reporting tools utilised in ALABAMA can be adapted for different RCTs investigating different health conditions. However, whilst we have established that utilising SystmOne for an RCT is feasible, researchers should consider allocating funding for a dedicated person with experience of working with the relevant EHR systems to ensure this work is carried out in a timely way. In theory, a similar methodology can be utilised in EHR platforms other than SystmOne, although close collaboration between EHR platform developers and trial teams is needed.

Health and personal data security is of paramount importance in routine care and in research. Since data security is a fundamental element of EHR systems, it seemed logical to utilise the data security measures already in place in established systems like SystmOne—another reason to try and embed research in routine EHR systems. The PRINCIPLE Trial (Platform Randomised Trial of Treatments in the Community for Epidemic and Pandemic Illnesses) was highly successful in its use of routinely collected data to identify potentially eligible patients with COVID-19 [17]. However, this trial was operating in a different legal context as the UK Secretary of State for Health and Social Care had issued NHS Digital with a notice to require NHS Digital to share confidential patient information with entitled organisations (under Regulation 3(4) of the NHS (Control of Patient Information Regulations) 2002 (COPI)) for COVID-19 purposes. Ways of achieving similar goals in the post-COVID-19 era are needed. The creation of the ALABAMA unit allowed patient data to be stored in a secure central repository (SystmOne) that could only be accessed by authorised members of the research team, thus providing a GDPR-compliant means of accessing patient data with minimal disturbance to participants and GPs.

Use of SystmOne functionality has had many additional advantages during delivery of the trial which increased efficiency, and quality of the trial data, allowing participants to be followed up remotely in real time. The functions developed for ALABAMA streamlined trial processes so that the trial was less reliant on laborious traditional data collection methods. Use of the SystmOne screening report allowed general practices to easily identify eligible participants within minutes, thus reducing the burden on participating general practices. Use of SystmOne also allowed delivery of some of the trial behaviour change interventions by alerting GPs to an updated allergy status when they prescribed an antibiotic to a participant whose penicillin allergy record had been updated following a negative allergy test result [14]. Updating primary care penicillin allergy records after penicillin allergy assessment has previously been identified as a problem [18]. In the ALABAMA trial, updating of allergy status was therefore facilitated through use of trial specific SNOMED CT concepts and a built-in process of communicating results via pop-up messages and “tasks” within SystmOne.

## Limitations

We successfully managed to embed SystmOne EHR into RCT delivery; however, this was a lengthy process that required appropriate funding and technical resources. This novel methodology was developed iteratively and tested at each stage, but it was not formally compared to more traditional trial methods as we lacked both funding and ethical approval for this. The ALABAMA processes required close monitoring and improvements throughout the trial; the research team worked closely with TPP who was available to troubleshoot if there were any issues with the CDK tools.

Data validity is important to consider when using routinely collected data as accuracy may also be impacted by the fact that the data captured in EHR is not validated [19]. EHR data is not primarily captured for research use, and it must be remembered there are therefore limitations of standardisation and quality of the data, for example data entry errors can occur. This was evident in the incomplete capturing of exclusion criteria in EHR and the absence of functionality within for SystmOne to generate and allocate unique trial identification numbers to trial participants. Additionally, there may be issues with incomplete or missing outcome data, for example the indication for antibiotic prescription was not always documented and therefore required some clinical interpretation based on what was recorded in the consultation. SystmOne data was largely limited to primary care, and so outcome data had to be supplemented through acquisition of secondary care data from a combination of secondary care records and data from NHS England databases (Hospital Episode Statistics and Office for National Statistics). Another issue encountered was incomplete or inaccurate data entry when using the CDK tools. During feasibility testing, we discovered that where the referral template to the ALABAMA unit was incompletely filled, or correct data sharing permissions not set, the referral resulted in data sharing restrictions and the research team were therefore unable to view all the relevant data. Once this was identified, SOPs/WI were updated and general practices sites retrained. This work was done through a research collaboration, but costs of obtaining bespoke reports would need to be built into funding of future EHR-based RCTs. Further work comparing use of EHR in RCT and traditional methods, in particular to assess the quality of outcome data collected and impact on workload, is needed.

## Conclusion

The ALABAMA trial has utilised functionality in SystmOne in a unique and novel way to allow more efficient use of an EHR system and routinely collected health data in the delivery of an RCT. We have demonstrated through collaboration between the ALABAMA research team and TPP that tools within SystmOne can be successfully adapted to trial processes and procedures. Provision of a bespoke ALABAMA unit provided GDPR complaint access to clinical data of trial participants allowing remote and real-time follow-up of participants. We believe that the SystmOne processes used in the ALABAMA trial can be adapted to be used in other primary care trials, improving trial efficiency and quality.

## Supplementary Information

Supplementary Material 1. Sect. 1: ALABAMA trial inclusion and exclusion criteria. Section 2: Supplementary figures. Figure S1. Process map and work instructions (WI) list for the Allergy antibiotic and microbial resistance (ALABAMA) trial (ClinicalTrials.gov: NCT04108637). Figure S2. The SystmOne ALABAMA electronic referral form used by general practitioners to refer patients into the ALABAMA unit and to confirm permission for sharing of the patient’s data. Figure S3. SystmOne template used in the ALABAMA trial to assign patients to a ‘caseload’ after randomisation. Figure S4. SystmOne template used in the ALABAMA trial to facilitate correct results and Read Code entry into a patients’ medical records. Section 3: Results. Table S1. ‘Y’ codes used to record penicillin allergy testing results. Section 4: Acknowledgements

## Abbreviations

ALABAMA: Allergy AntiBiotics And Microbial resistAnce trial
CKD: Clinical Development Kit
DPIA: Data Protection Impact Assessment
EHR: Electronic health record
GDPR: General Data Protection Regulation
GP: General practitioner
NHS: National Health Service
PPIE: Patient and public involvement and engagement
RCT: Randomised controlled trial
SOP: Standard operating procedures
TMG: Trial Management Group
TPP: The Phoenix Partnership (Leeds) Ltd.
TSC: Trial steering committee
WI: Working instructions
