# Supplementary material for: Utilising primary care electronic health records to deliver the ALABAMA randomised controlled trial of penicillin allergy assessment
Source: Trials. 2024 Oct 3;25:653. doi: 10.1186/s13063-024-08506-x (PMC11450982; doi:10.1186/s13063-024-08506-x)
Supplement: Supplementary file 1 — Supplementary Material 1. Sect. 1: ALABAMA trial inclusion and exclusion criteria. Section 2: Supplementary figures. Figure S1. Process map and work instructions (WI) list for the Allergy antibiotic and microbial resistance (ALABAMA) trial (ClinicalTrials.gov: NCT04108637). Figure S2. The SystmOne ALABAMA electronic referral form used by general practitioners to refer patients into the ALABAMA unit and to confirm permission for sharing of the patient’s data. Figure S3. SystmOne template used in the ALABAMA trial to assign patients to a ‘caseload’ after randomisation. Figure S4. SystmOne template used in the ALABAMA trial to facilitate correct results and Read Code entry into a patients’ medical records. Section 3: Results. Table S1. ‘Y’ codes used to record penicillin allergy testing results. Section 4: Acknowledgements [file 13063_2024_8506_MOESM1_ESM.docx]

**Utilising primary care electronic health records to deliver the ALABAMA randomised controlled trial of penicillin allergy assessment**

**Supplementary material**

**Section 1: ALABAMA trial inclusion and exclusion criteria**

Inclusion criteria:

- Participant is willing and able to give informed consent for participation in the trial
- Male or Female, aged 18 years or above
- Current penicillin allergy (or sensitivity) record of any kind in their electronic health record*
- Prescribed systemtic antibiotics in the previous 24 months**

**Patients who had been formally tested for penicillin allergy in the past and been found not to be penicillin allergic but still had a medical record indicating a penicillin allergy were also eligible for the trial.*

***Patients with a penicillin allergy record and a recent penicillin prescription were still considered to be be eligible because their allergy status required assessment and records correcting if necessary.*

Exclusion criteria:

- Life expectancy estimated <1 year by GP
- Unable to attend immunology clinic
- Unsuitable for entry into testing pathway because:
  - Allergy history consistent with anaphylaxis to penicillin
  - History of toxic epidermal necrolysis, Stevens-Johnson syndrome, Drug reaction with eosinophilia and systemic symptoms (DRESS) or any severe rash which blistered or needed hospital treatment, and acute generalised exanthematous pustulosis precipitated by a penicillin
  - Has been formally tested for penicillin allergy in the past and been found to be penicillin allergic
  - History of brittle/severe asthma or has had a course of steroids in the past 3 months for asthma or unstable coronary artery disease, or severe/poorly controlled skin conditions
  - Considered unsuitable for trial participation by the GP e.g. because of chaotic lifestyle
- Pregnant
- Breastfeeding mothers
- Currently taking beta blocker medication, and unable to temporarily withhold these on the day of penicillin allergy testing
- Currently taking (or recently taken) systemic steroids and unable to stop these for 10 days pre-testing
- Currently taking antihistamines and unable to temporarily withhold these for 72 hours pre-testing*
- General practitioners were also able to exclude vulnerable patients who are deemed to be unsuitable to participate for other reasons such as, but not limited to, terminal illness, reliability, mental illness, learning difficulties, anxiety, other family circumstances.

*Patients that are currently taking medicines with antihistamine properties that cannot be temporarily withheld, or patients with isolated dermographism, could be eligible to take part but would need prior discussion with the research team prior to consent.

**Section 2: Supplementary figures**

Figure S1. Process map and work instructions (WI) list for the Allergy antibiotic and microbial resistance (ALABAMA) trial (ClinicalTrials.gov: NCT04108637)


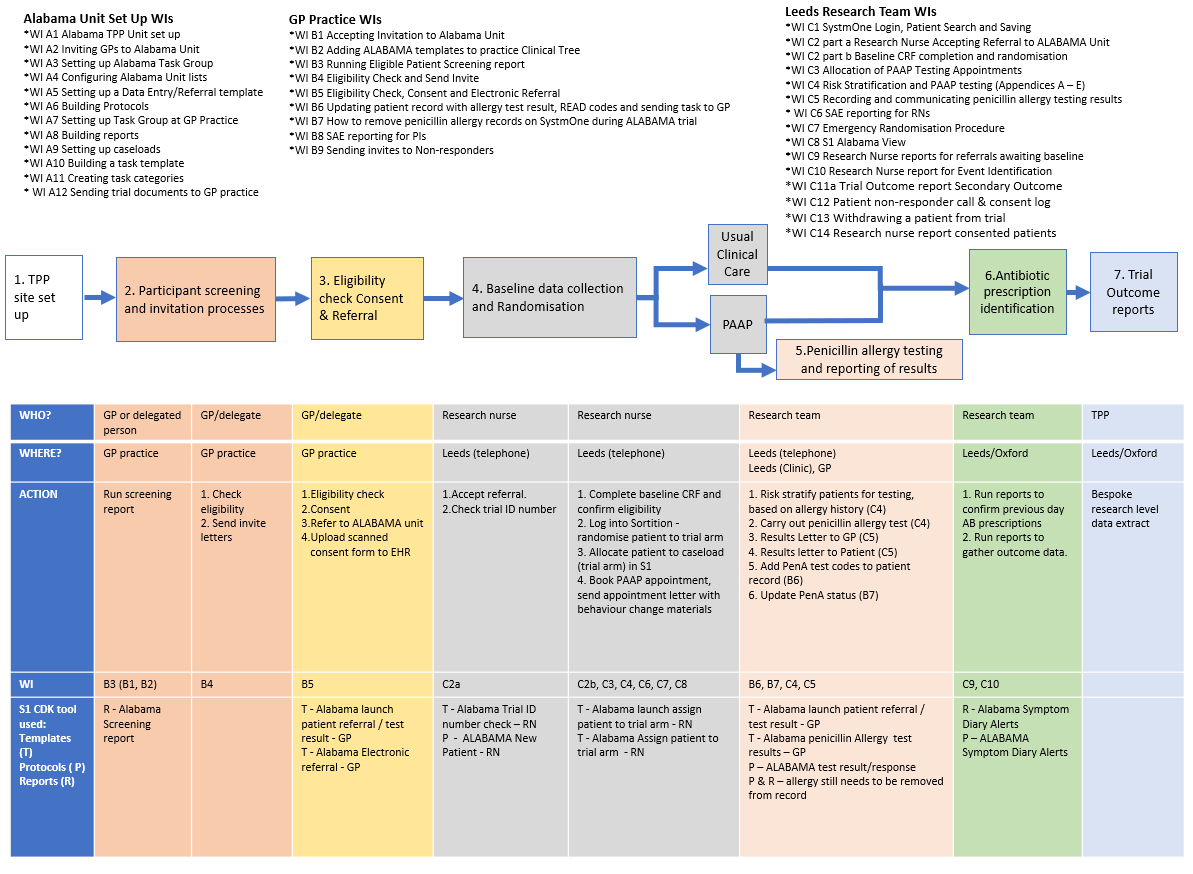

AB= Antibiotic, CDK = Clinical Decision Tool, CRF = Case report form, PAAP = Penicillin allergy assessment pathway (ALABAMA trial intervention), RN = Research nurse, UCC = usual clinical care, WI = Working Instructions

Figure S2. The SystmOne ALABAMA electronic referral form used by general practitioners to refer patients into the ALABAMA unit and to confirm permission for sharing of the patient’s data.


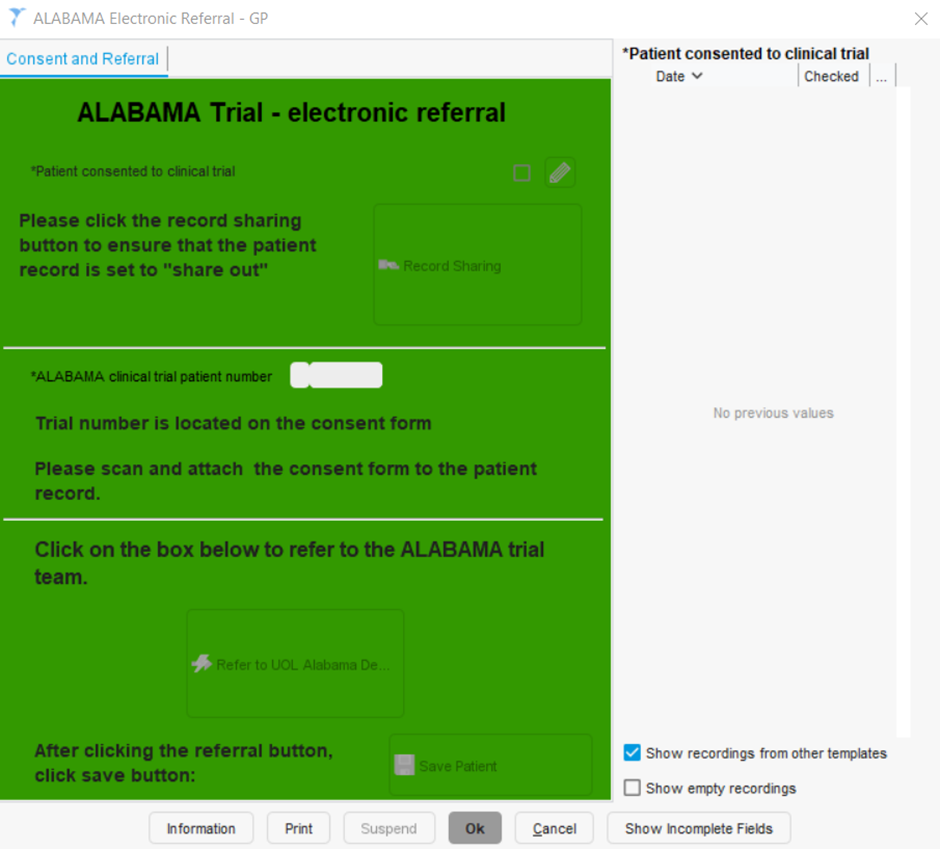


Figure S3. SystmOne template used in the ALABAMA trial to assign patients to a ‘caseload’ after randomisation


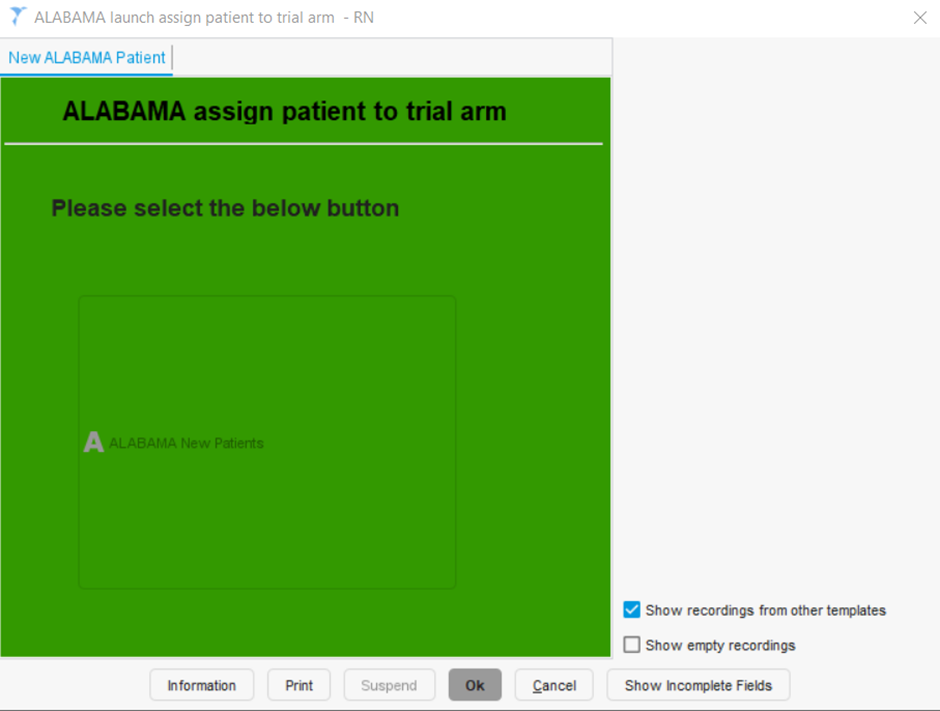


Figure S4. SystmOne template used in the ALABAMA trial to facilitate correct results and Read Code entry into a patients’ medical records


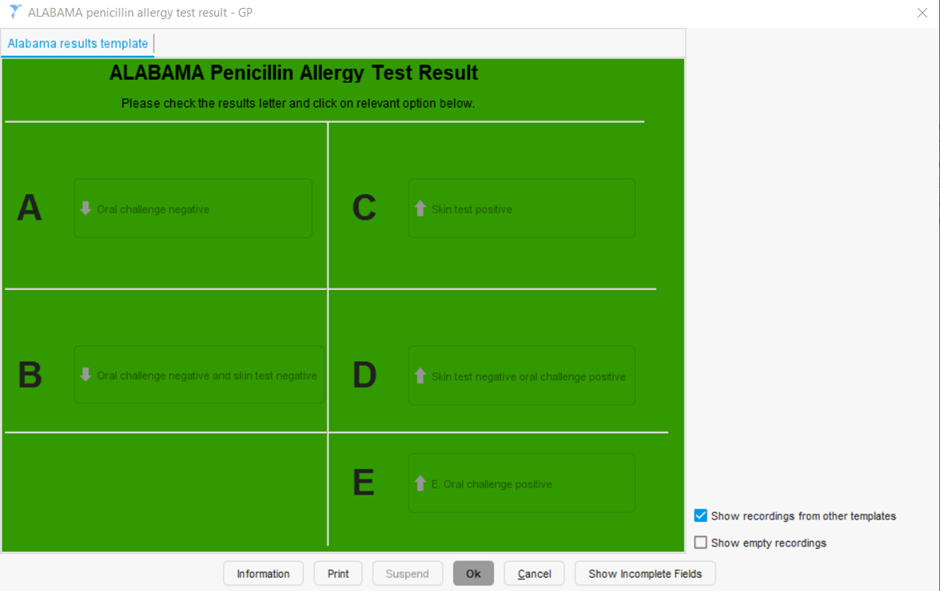


**Section 3: Results**

Participant screening and invitation processes

Monitoring performance of the screening report output during the feasibility phase identified that it captured a small number of participants who had allergies to clarithromycin and lansoprazole but not to penicillin. This was investigated and was found to be related to previous adverse reactions to *Helicobacter pylori* treatments, where lansoprazole, clarithromycin and amoxicillin had been prescribed together. If a GP had recorded a reaction to these drugs “and all related products” the participant would be identified by the screening report even if they did not have a penicillin allergy. Therefore, the screening report was amended to exclude patients that also had allergies to lansoprazole and clarithromycin. Following the feasibility phase of the trial, the screening report was amended to capture all antibiotics prescribed in the previous 24 months.

The screening report excluded some disorders listed in the exclusion criteria, but not all. We found Read (now SNOMED) codes for toxic epidermal necrolysis, Stevens-Johnson syndrome, Drug reaction with eosinophilia and systemic symptoms (DRESS) or any severe rash which blistered or needed hospital treatment, and acute generalised exanthematous pustulosis but if present, there would need to be a manual check to see if it had been precipitated by a penicillin.

The following exclusion criteria needed to be manually checked as data recording in the EHR records was not considered reliable enough:

- Life expectancy estimated <1 year by GP;
- Unable to attend hospital clinic where allergy testing takes place;
- Unsuitable for entry into testing pathway because:
  - Allergy history consistent with anaphylaxis to penicillin

Formally tested for penicillin allergy in the past and been found to be penicillin allergic

- - History of brittle/severe asthma or has had a course of steroids in the past 3 months for asthma or unstable coronary artery disease, or other severe/poorly controlled skin conditions
  - Considered unsuitable for trial participation by the GP e.g. because of chaotic lifestyle
- Pregnant
- Breastfeeding mothers
- Currently taking beta blocker medication, and unable to temporarily withhold these on the day of penicillin allergy testing
- Currently taking (or recently taken) systemic steroids and unable to stop these for 10 days pre-testing
- Currently taking antihistamines or taking an antihistamine containing drug, and unable to temporarily withhold these for 72 hours pre-testing

| **Code** | **Descriptor** |
| --- | --- |
| Y1b3d | Oral penicillin challenge test negative |
| Y1b3c | Oral penicillin challenge test positive |
| Y1b39 | Skin prick test for penicillin negative |
| Y1b38 | Skin prick test for penicillin positive |

Penicillin allergy testing and reporting of results

Table S1. 'Y' codes used to record penicillin allergy testing results.

**Section 4: Acknowledgements**

The authors would like to acknowledge the contributions of the ALABAMA research team to the trial:, Sammiya Ahmed, Angel Aspinwall, Rebecca Bestwick, Rebecca Burnham, Emma Carter, Jem Chalk, Sam Charlton, Johanna Cook, Kate Corfield, Mina Davoudianfar, Naila Dracup, Claire Forrest, Alison Haigh, Daniel Howdon, Claire Jarman, Honorine Jobain, Dave Judge, Gary Lamont, Tommaso Malavenda, Cheridan Morgan, Ruben Mujica-Mota, Alina Negut, Michael Padden, Meena Patil, Daniel Plotkin, Neil Powell, Joy Rahmen, Claire Reidy, Caity Roleston, Ravishankur Sagur, Gaushiya Saiyad, Razan Saman, Gaye Sheerman Chase, Bethany Shinkins, Samuel Skuse, Wioletta Sobacka, Robert White, Nicola Window, Judy Wright, Miaoqing Yang, Ly-Mee Yu.

We would also like to thank the TPP product specialists for their technical support throughout the trial.
